# Crystal structure and Hirshfeld surface analysis of [2-(1*H*-benzimidazol-2-yl-κ*N* ^3^)aniline-κ*N*]di­chlorido­zinc(II) *N*,*N*-di­methyl­formamide monosolvate

**DOI:** 10.1107/S2056989021003649

**Published:** 2021-04-09

**Authors:** Mohd Muslim, Md. Serajul Haque Faizi, Arif Ali, Mohd Afzal, Musheer Ahmad, Necmi Dege, Ashraf Mashrai

**Affiliations:** a Department of Applied Chemistry, ZHCET, Faculty of Engineering and Technology, Aligarh Muslim University, Aligarh, 202002, (UP), India; b PG Department of Chemistry, Langat Singh College, B.R.A. Bihar University, Muzaffarpur, Bihar 842001, India; c Department of Chemistry, College of Science, King Saud University, Riyadh-11451, Kingdom of, Saudi Arabia; d Ondokuz Mayis University, Arts and Sciences Faculty, Department of Physics, Atakum 55139 Samsun, Turkey; e Department of Pharmacy, University of Science and Technology, Ibb Branch, Yemen

**Keywords:** crystal structure, benzimidazole, aniline, bidentate ligand, heterocyclic, zinc(II) complex

## Abstract

The title compound, [ZnCl_2_(C_13_H_11_N_3_)]·C_3_H_7_NO, crystallized in the monoclinic crystal system in space group *P*2_1_/*n*. The asymmetric unit contains one neutral complex mol­ecule, which consists of a zinc ion, a bidentate ligand, and two chlorido ligands with di­methyl­formamide monosolvate. The ligand has two moieties, a benzimidazole and an aniline group. The benzimidazole and aniline planes are not coplanar, subtending a dihedral angle of 18.24 (8)°. The Zn(II) ion shows distorted tetra­hedral geometry, being coordinated by an imidazole N atom, the aniline N atom, and two chlorido ligands. The packing features N—H⋯O, N—H⋯Cl, C—H⋯Cl hydrogen bonding.

## Abstract 2

The asymmetric unit of the title complex contains one independent neutral complex mol­ecule, which consists of one zinc(II) ion, one bidentate ligand, and two chlorido ligands. The ligand consists of two moieties: benzimidazole and aniline. The Zn(II) ion adopts a distorted tetra­hedral coordination geometry. A Hirshfeld surface analysis was performed to qu­antify the inter­molecular inter­actions and help understand the overall packing nature of the title compound.

## Chemical context   

Benzimidazoles as organic ligands have attracted inter­est with regard to the synthesis of metal–organic frameworks, not only because of their coordination abilities to metal ions, but also their significant potential applications in biological systems (Ahmad & Bharadwaj; 2013 ▸; Sharma *et al.*, 2016 ▸; Gu *et al.*, 2017 ▸). Benzimidazole compounds and their metal complexes have been found to show diverse biological activity (Podunavac-Kuzmanovic & Cvetkovic, 2010 ▸), including inhibition against enteroviruses (Xue *et al.*, 2011 ▸) and potent anti­tumor activity (Galarce *et al.*, 2008 ▸). The bidentate ligand 2-(1*H*-benzo[*d*]imidazol-2-yl) aniline (*L*) has been used to prepare a series of mononuclear transition-metal complexes with halide anions as the active leaving group in our catalytic research. In this work, a mononuclear zinc complex Zn*L*Cl_2_ is reported. Zinc complexes bearing various ancillary ligands have been applied in the catalysis of the copolymerization of cyclo­hexene oxide and CO_2_ (Kember *et al.*, 2009 ▸).
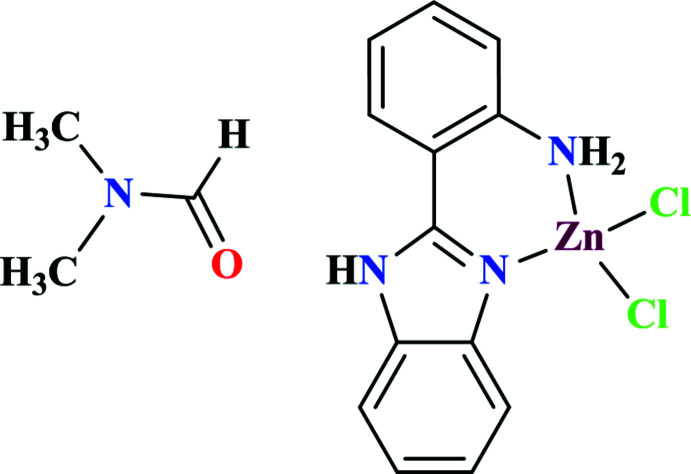


## Structural commentary   

**Figure 1:**
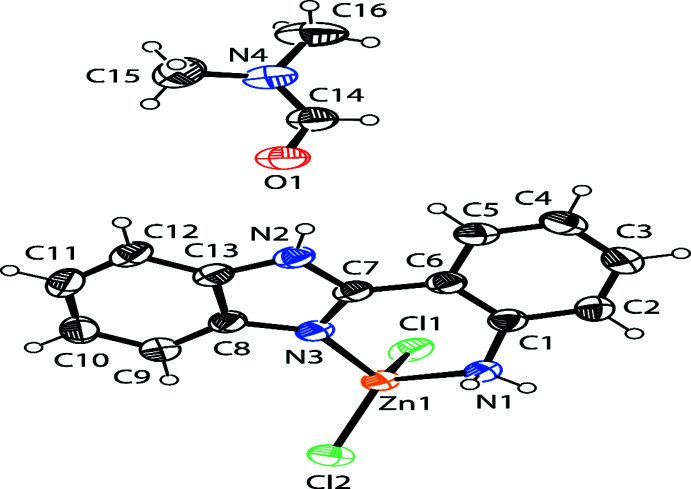
A view of the title complex with the atom labeling and displacement ellipsoids drawn at the 40% level.

The asymmetric unit of the title complex (Fig. 1 ▸) contains one neutral complex mol­ecule, which consists of one central zinc ion, one bidentate ligand, and two chlorido ligands with di­methyl­formamide solvent. The two ligand moieties, benzimidazole and aniline, are not coplanar structure, subtending a dihedral angle of 18.24 (8)°. The C1—N1 and C7—N2 bond lengths are 1.449 (2) and 1.335 (2) Å, respectively. The complex is a four-coordinated system by one imidazole nitro­gen atom N2, one aniline nitro­gen atom N1, and two chlorido ligands. The distances from the zinc(II) ion to the coordinating atoms are all in the expected ranges. The bond angles around the zinc(II) atom are in the range 88.64 (7) to 118.57 (3)°, of which the smallest angle N1—Zn1—N2 is formed by the two nitro­gen atoms from the bidentate ligand.

## Supra­molecular features   

**Figure 2:**
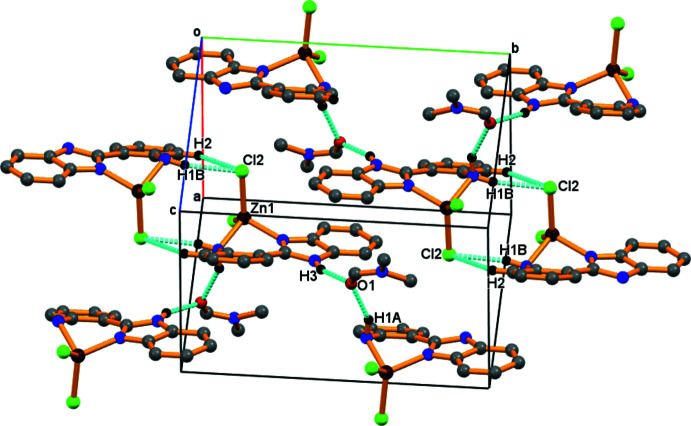
A packing view approximately along [10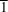] of the title complex. Hydrogen atoms are omitted for clarity.

**Figure 3:**
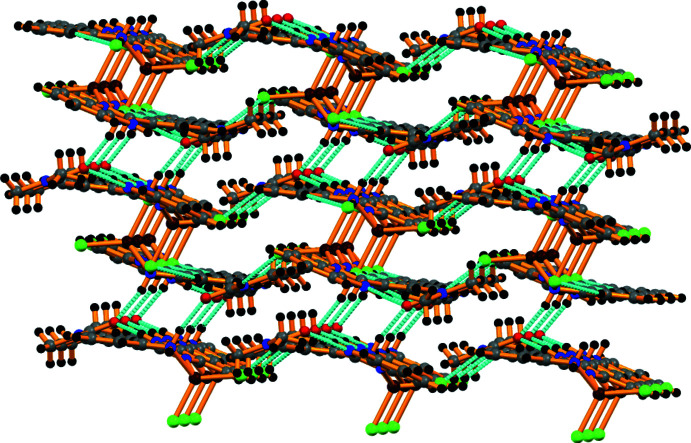
Supra­molecular view along the *b* axis of the crystal structure of the title complex formed through C—H⋯π, hydrogen-bonding, and other weak inter­actions.

In the crystal, mol­ecules are linked by N—H⋯Cl hydrogen bonds (Table 1 ▸, Fig. 2 ▸), forming sheets propagating along the *b*-axis direction (Fig. 3 ▸).

## Hirshfeld Surface analysis   

**Figure 4:**
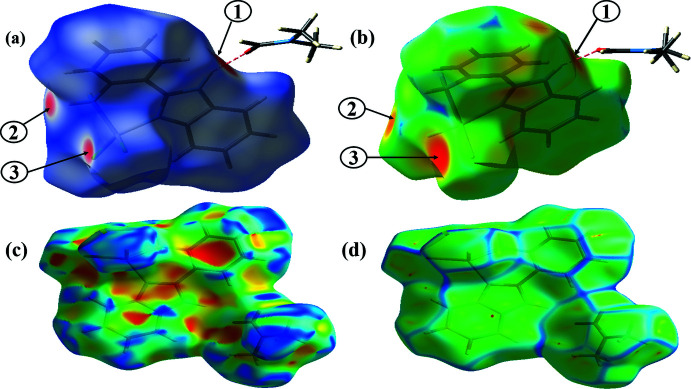
The Hirshfeld surface of the title complex mapped over (*a*) *d*_norm_, (*b*) *d_e_*, (*c*) shape-index, and (*d*) curvedness. Red spots 1,2, and 3 in (*a*) and (*b*) correspond to N—H⋯O, N—H⋯Cl, and C—H⋯Cl hydrogen bonds.

A Hirshfeld surface analysis (Spackman & Jayatilaka, 2009 ▸) was undertaken and the associated two-dimensional fingerprint plots (McKinnon, *et al.*, 2007 ▸) generated using *Crystal Explorer* (Turner *et al.*, 2017 ▸) to investigate the inter­molecular inter­actions and surface morphology of the crystal structure. The Hirshfeld surface mapped over *d*_norm_ in the color range (^_^0.464 to 1.340 a.u.) from red (shorter than the sum of the van der Waals radii) and white to blue (longer distance than the sum of the van der Waals radii). The bright red spot on the *d*_norm_ surface (Fig. 4 ▸ *a*) indicates the N—H⋯O hydrogen bonding. C—H⋯Cl contacts are evident as distinct circular depressions (red spots) and other visible spots on the *d*_norm_ surface (Fig. 4 ▸ *a*) are due to H⋯H contacts. The surfaces of the title complex were also mapped over *d*_e_ (0.834 to 2.650 Å), shape-index (−1.0000 to 1.0000 Å), and curvedness (−4.0000 to 0.4000 Å) in the given ranges. The flat green region on the *d*_e_ surface represents similar contact distances (Fig. 4 ▸ *b*). The pattern of red and blue regions in the shape-index surface is characteristic of ring carbon atoms of the mol­ecule inside the surface. The shape of the blue outline on the curved surface shown in Fig. 4 ▸ *d* is evidence of the flat region toward the bottom of both sides of the mol­ecules.

**Figure 5:**
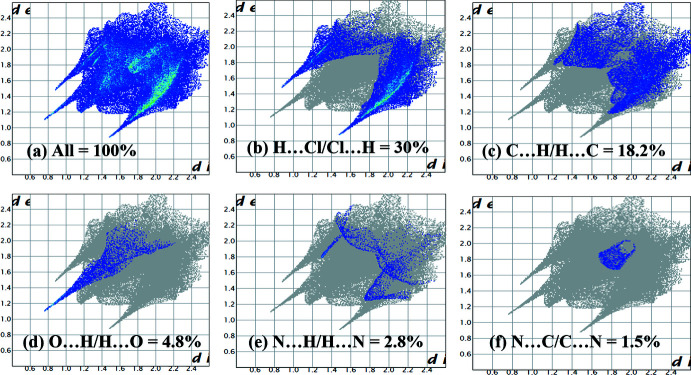
(*a*) A full two-dimensional fingerprint plot of the title complex, and delineated into (*b*) H⋯Cl/Cl⋯H (30%), (*c*) C⋯H/H⋯C (18.2%), and (*d*) O⋯H/H⋯O (4.8%) contacts, which are the major inter­actions present in the crystal structure.

Five types of major inter­actions in the crystal structure (H⋯Cl = 30%, C⋯H = 18.2%, O⋯H = 4.8%, N⋯H = 2.8%, N⋯C = 1.5%) are shown in the two-dimensional fingerprint plots (Fig. 5 ▸). The inter­action order (H⋯Cl)> (C⋯H)> (O⋯H)> (N⋯H)> (N⋯C) of *d*_norm_ on the 2D fingerprint plot represents the nature of packing in the title crystal structure (Muslim *et al.*, 2020 ▸). The pattern of inter­molecular inter­actions (H⋯Cl/Cl⋯H, C⋯H/H⋯C, O⋯H/H⋯O, N⋯H/H⋯O, and N⋯C/C⋯N) governs the overall packing and qu­anti­fies the contribution of the non-covalent inter­action (C—H⋯Cl) to the extended supra­molecular network (Seth *et al.*, 2011 ▸; Seth, 2013 ▸; Manna *et al.*, 2012 ▸; Mitra *et al.*, 2014 ▸).

## Database survey   

A search of the Cambridge Structural Database (CSD, version 5.39; Groom *et al.*, 2016 ▸) gave thirteen hits for the [2-(1*H*-benzimidazol-2-yl]aniline)zinc(II) moiety. Two compounds whose structures are very similar to that of the title compound are [2-(1*H*-benzimidazol-2-yl)aniline]di­chlorido­zinc(II) (AWOLEE; Eltayeb *et al.*, 2011*a* ▸) in which the di­methyl­formamide solvent is absent and di­chloro-[2-(1-methyl-1*H*-benzimidazol-2-yl)aniline]zinc(II) (ILELIW; Zhou *et al.*, 2016 ▸) in which the NH group is replaced by an N—CH_3_ group. Zinc compounds with different ligands include (2-{[2-(1*H*-benzimidazol-2-yl-κ*N* ^3^)phen­yl]imino­methyl-κ*N*}-5-meth­ylphenolato-κ*O*)chlorido­zinc(II) (AYINEC; Eltayeb *et al.*, 2011*b* ▸) in which the zinc atom is surrounded by two imine nitro­gen, one phenolic oxygen and one chlorine atoms. Other complexes include bis­{N-[2-(1-butyl-5-nitro-1*H*-benzimidazol-2-yl)phen­yl]-4- methyl­benzene­sulfonamidato}zinc(II) with an unknown solvate (BUXDIJ; Burlov *et al.*, 2016 ▸) and bis­{4-methyl-*N*-[2-(5-nitro-1-propyl-1*H*-benzimidazole-2-yl)phen­yl]benzene­sulfonamidato}zinc(II) chloro­form solvate (BUXDOP; Burlov *et al.*, 2016 ▸), bis­{μ-[2-(5-amino-1-propyl-1*H*-benzimidazole-2-yl)phen­yl](4-methyl­benzene-1-sulfon­yl)amido}­bis­(pivalato)dizinc aceto­nitrile ethanol solvate dihydrate (EDOVUR; Nikolaevskii *et al.*, 2014 ▸), bis­(μ_2_-3-{[2-(1*H*-benzimidazole-2-yl)phen­yl]carbonoimido­yl}benzene-1,2-diolato)dizinc(II) ethanol solvate (GABVUD; Wang *et al.*, 2016 ▸), (acetato-*O*,*O*′)-[2-({[2-(1*H*-benzimidazole-2-yl)phen­yl]imino}­meth­yl)-5-(di­ethyl­amino)­phenolato]zinc(II) iso­pro­p­anol solvate (IKOYUE; Liao *et al.*, 2016 ▸).

## Synthesis and crystallization   

A mixture of 2-(2-amino­phenyl­benzimidazole) (0.05 g, 0.14 mmol) and ZnCl_2_·4H_2_O (0.125 g, 0.4 mmol) was dissolved in 5 ml of di­methyl­formamide (DMF) and then sealed in a Teflon-lined autoclave and heated under autogenous pressure to 453 K for 2 d and then allow to cool to room temperature at the rate of 1 K per minute. The resulting solution was filtered and kept for slow evaporation. After one week, block-shaped colorless crystals suitable for single-crystal X-ray diffraction data collection were obtained.

## Refinement   

Crystal data, data collection and structure refinement details are summarized in Table 2 ▸. Hydrogen atoms were positioned geometrically (N—H = 0.86–0.89, C—H = 0.93–0.96 Å) included with *U*_iso_(H) = 1.2*U*_eq_(N, C) or 1.5*U*_eq_(C-meth­yl).

## Supplementary Material

Crystal structure: contains datablock(s) I. DOI: 10.1107/S2056989021003649/ex2042sup1.cif

Structure factors: contains datablock(s) I. DOI: 10.1107/S2056989021003649/ex2042Isup4.hkl

CCDC reference: 2033195

Additional supporting information:  crystallographic information; 3D view; checkCIF report

## Figures and Tables

**Table 1:** Hydrogen-bond geometry (Å, °)

*D*—H⋯*A*	*D*—H	H⋯*A*	*D*⋯*A*	*D*—H⋯*A*
N1—H1*A*⋯O1^i^	0.89	2.11	2.923 (2)	152
N1—H1*B*⋯Cl2^ii^	0.89	2.48	3.3592 (17)	170
N3—H3⋯O1	0.86	1.99	2.807 (2)	157
C2—H2⋯Cl2^ii^	0.93	2.93	3.734 (2)	145
C14—H14⋯Cl1^iii^	0.93	2.95	3.836 (3)	160

Symmetry codes: (i) -x+{\script{1\over 2}}, y+{\script{1\over 2}}, -z+{\script{1\over 2}}; (ii) -x+1, -y+2, -z+1; (iii) x+{\script{1\over 2}}, -y+{\script{3\over 2}}, z-{\script{1\over 2}}.

**Table 2:** Experimental details

Crystal data	
Chemical formula	[ZnCl_2_(C_13_H_11_N_3_)]·C_3_H_7_NO
*M*_r_	418.61
Crystal system, space group	Monoclinic, *P*2_1_/*n*
Temperature (K)	296
*a*, *b*, *c* (Å)	10.9394 (9), 13.3041 (7), 13.1665 (11)
β (°)	106.140 (7)
*V* (Å^3^)	1840.7 (2)
*Z*	4
Radiation type	Mo *K*α
μ (mm^−1^)	1.64
Crystal size (mm)	0.60 × 0.50 × 0.39

Data collection	
Diffractometer	Stoe IPDS 2
Absorption correction	Integration (*X-RED32*; Stoe & Cie, 2002 ▸)
*T*_min_, *T*_max_	0.460, 0.567
No. of measured, independent and observed [*I* > 2σ(*I*)] reflections	19616, 5643, 3891
*R*_int_	0.036
(sin θ/λ)_max_ (Å^−1^)	0.717

Refinement	
*R*[*F* ^2^ > 2σ(*F* ^2^)], *wR*(*F* ^2^), *S*	0.037, 0.097, 1.04
No. of reflections	5643
No. of parameters	219
H-atom treatment	H-atom parameters constrained
Δρ_max_, Δρ_min_ (e Å^−3^)	0.34, −0.62

Computer programs: *X-AREA* (Stoe & Cie, 2002 ▸), *SHELXT* (Sheldrick, 2015*a* ▸), *SHELXL2018/3* (Sheldrick, 2015*b* ▸), *ORTEP-3 for Windows* (Farrugia, 2012 ▸), and *XP* in *SHELXTL* (Sheldrick, 2008 ▸).

## supplementary crystallographic information

### Crystal data

**Table:**

[ZnCl_2_(C_13_H_11_N_3_)]·C_3_H_7_NO	*F*(000) = 856
*M**_r_* = 418.61	*D*_x_ = 1.511 Mg m^−^^3^
Monoclinic, *P*2_1_/*n*	Mo *K*α radiation, λ = 0.71073 Å
*a* = 10.9394 (9) Å	Cell parameters from 22582 reflections
*b* = 13.3041 (7) Å	θ = 1.5–31.0°
*c* = 13.1665 (11) Å	µ = 1.64 mm^−^^1^
β = 106.140 (7)°	*T* = 296 K
*V* = 1840.7 (2) Å^3^	Prism, yellow
*Z* = 4	0.60 × 0.50 × 0.39 mm

### Data collection

**Table:**

STOE IPDS 2 diffractometer	5643 independent reflections
Radiation source: sealed X-ray tube, 12 x 0.4 mm long-fine focus	3891 reflections with *I* > 2σ(*I*)
Plane graphite monochromator	*R*_int_ = 0.036
Detector resolution: 6.67 pixels mm^-1^	θ_max_ = 30.7°, θ_min_ = 2.2°
rotation method scans	*h* = −15→15
Absorption correction: integration (X-RED32; Stoe & Cie, 2002)	*k* = −17→18
*T*_min_ = 0.460, *T*_max_ = 0.567	*l* = −18→18
19616 measured reflections	

### Refinement

**Table:**

Refinement on *F*^2^	0 restraints
Least-squares matrix: full	Hydrogen site location: inferred from neighbouring sites
*R*[*F*^2^ > 2σ(*F*^2^)] = 0.037	H-atom parameters constrained
*wR*(*F*^2^) = 0.097	*w* = 1/[σ^2^(*F*_o_^2^) + (0.0502*P*)^2^ + 0.0362*P*] where *P* = (*F*_o_^2^ + 2*F*_c_^2^)/3
*S* = 1.04	(Δ/σ)_max_ = 0.001
5643 reflections	Δρ_max_ = 0.34 e Å^−^^3^
219 parameters	Δρ_min_ = −0.62 e Å^−^^3^

### Special details

**Table:**

Geometry. All esds (except the esd in the dihedral angle between two l.s. planes) are estimated using the full covariance matrix. The cell esds are taken into account individually in the estimation of esds in distances, angles and torsion angles; correlations between esds in cell parameters are only used when they are defined by crystal symmetry. An approximate (isotropic) treatment of cell esds is used for estimating esds involving l.s. planes.

### Fractional atomic coordinates and isotropic or equivalent isotropic displacement parameters (Å^2^)

**Table:**

	*x*	*y*	*z*	*U*_iso_*/*U*_eq_	
Zn1	0.40345 (2)	0.83177 (2)	0.52883 (2)	0.04912 (8)	
Cl2	0.61440 (5)	0.84302 (4)	0.60104 (5)	0.06630 (16)	
Cl1	0.27561 (6)	0.87107 (5)	0.62724 (5)	0.07198 (17)	
O1	0.41861 (16)	0.45845 (14)	0.17389 (14)	0.0726 (5)	
N1	0.35146 (16)	0.90600 (12)	0.38489 (13)	0.0502 (4)	
H1A	0.267634	0.900599	0.357590	0.060*	
H1B	0.369650	0.970981	0.395980	0.060*	
N3	0.37815 (15)	0.58840 (12)	0.32936 (13)	0.0504 (4)	
H3	0.388210	0.563774	0.271825	0.060*	
N2	0.36342 (15)	0.70047 (12)	0.44985 (13)	0.0476 (3)	
N4	0.5564 (2)	0.35468 (17)	0.12569 (17)	0.0691 (5)	
C7	0.38739 (18)	0.68676 (14)	0.35677 (15)	0.0455 (4)	
C1	0.41353 (18)	0.86859 (16)	0.30855 (15)	0.0480 (4)	
C13	0.34988 (17)	0.53440 (15)	0.40928 (16)	0.0486 (4)	
C8	0.33936 (18)	0.60573 (14)	0.48466 (16)	0.0479 (4)	
C6	0.42738 (18)	0.76471 (15)	0.29345 (15)	0.0482 (4)	
C12	0.3365 (2)	0.43147 (16)	0.42430 (19)	0.0590 (5)	
H12	0.345224	0.384308	0.374641	0.071*	
C2	0.4613 (2)	0.93761 (18)	0.25020 (18)	0.0582 (5)	
H2	0.452680	1.005994	0.261168	0.070*	
C9	0.3122 (2)	0.57631 (17)	0.57798 (18)	0.0588 (5)	
H9	0.304769	0.623164	0.628334	0.071*	
C10	0.2969 (2)	0.47426 (18)	0.5923 (2)	0.0652 (6)	
H10	0.277820	0.452174	0.653141	0.078*	
C11	0.3097 (2)	0.40357 (18)	0.5165 (2)	0.0658 (6)	
H11	0.299785	0.335685	0.529071	0.079*	
C5	0.4888 (2)	0.73555 (19)	0.21791 (18)	0.0610 (5)	
H5	0.499020	0.667438	0.206631	0.073*	
C14	0.5136 (2)	0.4429 (2)	0.1430 (2)	0.0673 (6)	
H14	0.558734	0.498851	0.131104	0.081*	
C3	0.5212 (2)	0.9066 (2)	0.1763 (2)	0.0674 (6)	
H3A	0.552587	0.953741	0.137785	0.081*	
C4	0.5345 (2)	0.8051 (2)	0.1598 (2)	0.0694 (6)	
H4	0.574124	0.783670	0.109566	0.083*	
C15	0.4900 (4)	0.2641 (2)	0.1419 (3)	0.0947 (9)	
H15A	0.458046	0.272789	0.202229	0.142*	
H15B	0.547714	0.208200	0.153689	0.142*	
H15C	0.420336	0.251482	0.080351	0.142*	
C16	0.6699 (3)	0.3452 (3)	0.0881 (3)	0.1086 (13)	
H16A	0.700566	0.410905	0.077503	0.163*	
H16B	0.648854	0.308946	0.022441	0.163*	
H16C	0.734825	0.309531	0.139603	0.163*	

### Atomic displacement parameters (Å^2^)

**Table:**

	*U*^11^	*U*^22^	*U*^33^	*U*^12^	*U*^13^	*U*^23^
Zn1	0.05561 (14)	0.04018 (13)	0.05557 (14)	−0.00002 (10)	0.02208 (10)	−0.00610 (10)
Cl2	0.0557 (3)	0.0485 (3)	0.0914 (4)	0.0007 (2)	0.0149 (3)	−0.0107 (3)
Cl1	0.0790 (4)	0.0697 (4)	0.0814 (4)	0.0010 (3)	0.0458 (3)	−0.0117 (3)
O1	0.0641 (9)	0.0727 (11)	0.0876 (11)	−0.0039 (8)	0.0320 (9)	−0.0269 (9)
N1	0.0536 (9)	0.0383 (8)	0.0625 (10)	0.0024 (7)	0.0226 (8)	−0.0004 (7)
N3	0.0548 (9)	0.0409 (9)	0.0557 (9)	−0.0016 (7)	0.0155 (7)	−0.0098 (7)
N2	0.0513 (8)	0.0383 (8)	0.0561 (9)	0.0002 (7)	0.0198 (7)	−0.0054 (7)
N4	0.0641 (11)	0.0695 (13)	0.0731 (12)	0.0084 (10)	0.0178 (10)	−0.0187 (10)
C7	0.0450 (9)	0.0381 (10)	0.0536 (10)	0.0006 (7)	0.0138 (8)	−0.0064 (8)
C1	0.0458 (9)	0.0458 (10)	0.0531 (10)	0.0001 (8)	0.0152 (8)	−0.0010 (8)
C13	0.0411 (9)	0.0405 (10)	0.0621 (11)	0.0009 (7)	0.0108 (8)	−0.0044 (8)
C8	0.0442 (9)	0.0393 (9)	0.0612 (11)	−0.0011 (7)	0.0162 (8)	−0.0023 (8)
C6	0.0462 (9)	0.0459 (10)	0.0533 (10)	−0.0012 (8)	0.0152 (8)	−0.0047 (8)
C12	0.0551 (11)	0.0406 (11)	0.0781 (14)	−0.0027 (9)	0.0131 (10)	−0.0075 (10)
C2	0.0593 (12)	0.0504 (12)	0.0690 (13)	−0.0024 (9)	0.0245 (10)	0.0031 (10)
C9	0.0626 (12)	0.0531 (12)	0.0657 (12)	0.0021 (10)	0.0259 (10)	0.0019 (10)
C10	0.0634 (13)	0.0587 (14)	0.0778 (15)	−0.0017 (11)	0.0269 (11)	0.0136 (11)
C11	0.0586 (12)	0.0436 (11)	0.0936 (17)	−0.0033 (10)	0.0185 (12)	0.0092 (11)
C5	0.0686 (13)	0.0557 (13)	0.0651 (12)	0.0001 (10)	0.0295 (11)	−0.0092 (10)
C14	0.0619 (13)	0.0669 (15)	0.0772 (14)	−0.0053 (11)	0.0262 (11)	−0.0206 (12)
C3	0.0711 (14)	0.0680 (15)	0.0716 (14)	−0.0072 (12)	0.0340 (12)	0.0047 (12)
C4	0.0759 (15)	0.0767 (16)	0.0670 (14)	−0.0022 (13)	0.0388 (12)	−0.0059 (12)
C15	0.122 (3)	0.0664 (18)	0.098 (2)	0.0083 (18)	0.0341 (19)	−0.0014 (16)
C16	0.0760 (19)	0.124 (3)	0.134 (3)	0.0120 (18)	0.0440 (19)	−0.051 (2)

### Geometric parameters (Å, º)

**Table:**

Zn1—N2	2.0177 (16)	C6—C5	1.401 (3)
Zn1—N1	2.0715 (17)	C12—C11	1.376 (3)
Zn1—Cl1	2.2171 (6)	C12—H12	0.9300
Zn1—Cl2	2.2432 (7)	C2—C3	1.379 (3)
O1—C14	1.234 (3)	C2—H2	0.9300
N1—C1	1.449 (2)	C9—C10	1.387 (3)
N1—H1A	0.8900	C9—H9	0.9300
N1—H1B	0.8900	C10—C11	1.406 (4)
N3—C7	1.354 (2)	C10—H10	0.9300
N3—C13	1.378 (3)	C11—H11	0.9300
N3—H3	0.8600	C5—C4	1.380 (3)
N2—C7	1.335 (2)	C5—H5	0.9300
N2—C8	1.391 (2)	C14—H14	0.9300
N4—C14	1.307 (3)	C3—C4	1.382 (4)
N4—C15	1.453 (4)	C3—H3A	0.9300
N4—C16	1.465 (3)	C4—H4	0.9300
C7—C6	1.471 (3)	C15—H15A	0.9600
C1—C2	1.389 (3)	C15—H15B	0.9600
C1—C6	1.410 (3)	C15—H15C	0.9600
C13—C12	1.397 (3)	C16—H16A	0.9600
C13—C8	1.401 (3)	C16—H16B	0.9600
C8—C9	1.398 (3)	C16—H16C	0.9600
			
N2—Zn1—N1	88.64 (7)	C13—C12—H12	121.8
N2—Zn1—Cl1	115.06 (5)	C3—C2—C1	121.2 (2)
N1—Zn1—Cl1	111.40 (5)	C3—C2—H2	119.4
N2—Zn1—Cl2	109.06 (5)	C1—C2—H2	119.4
N1—Zn1—Cl2	110.14 (5)	C10—C9—C8	117.2 (2)
Cl1—Zn1—Cl2	118.57 (3)	C10—C9—H9	121.4
C1—N1—Zn1	114.12 (12)	C8—C9—H9	121.4
C1—N1—H1A	108.7	C9—C10—C11	121.2 (2)
Zn1—N1—H1A	108.7	C9—C10—H10	119.4
C1—N1—H1B	108.7	C11—C10—H10	119.4
Zn1—N1—H1B	108.7	C12—C11—C10	122.2 (2)
H1A—N1—H1B	107.6	C12—C11—H11	118.9
C7—N3—C13	108.43 (16)	C10—C11—H11	118.9
C7—N3—H3	125.8	C4—C5—C6	121.8 (2)
C13—N3—H3	125.8	C4—C5—H5	119.1
C7—N2—C8	106.38 (16)	C6—C5—H5	119.1
C7—N2—Zn1	121.43 (13)	O1—C14—N4	125.6 (3)
C8—N2—Zn1	130.43 (13)	O1—C14—H14	117.2
C14—N4—C15	120.1 (2)	N4—C14—H14	117.2
C14—N4—C16	121.0 (3)	C2—C3—C4	119.7 (2)
C15—N4—C16	118.9 (3)	C2—C3—H3A	120.2
N2—C7—N3	110.96 (17)	C4—C3—H3A	120.2
N2—C7—C6	126.08 (17)	C5—C4—C3	119.8 (2)
N3—C7—C6	122.84 (17)	C5—C4—H4	120.1
C2—C1—C6	119.87 (18)	C3—C4—H4	120.1
C2—C1—N1	118.52 (18)	N4—C15—H15A	109.5
C6—C1—N1	121.60 (17)	N4—C15—H15B	109.5
N3—C13—C12	132.33 (19)	H15A—C15—H15B	109.5
N3—C13—C8	105.54 (17)	N4—C15—H15C	109.5
C12—C13—C8	122.1 (2)	H15A—C15—H15C	109.5
N2—C8—C9	130.48 (18)	H15B—C15—H15C	109.5
N2—C8—C13	108.66 (17)	N4—C16—H16A	109.5
C9—C8—C13	120.83 (19)	N4—C16—H16B	109.5
C5—C6—C1	117.58 (19)	H16A—C16—H16B	109.5
C5—C6—C7	118.94 (18)	N4—C16—H16C	109.5
C1—C6—C7	123.39 (17)	H16A—C16—H16C	109.5
C11—C12—C13	116.5 (2)	H16B—C16—H16C	109.5
C11—C12—H12	121.8		
			
C8—N2—C7—N3	0.9 (2)	N1—C1—C6—C7	−3.5 (3)
Zn1—N2—C7—N3	167.25 (13)	N2—C7—C6—C5	158.9 (2)
C8—N2—C7—C6	−175.14 (18)	N3—C7—C6—C5	−16.7 (3)
Zn1—N2—C7—C6	−8.8 (3)	N2—C7—C6—C1	−17.5 (3)
C13—N3—C7—N2	−1.5 (2)	N3—C7—C6—C1	166.93 (18)
C13—N3—C7—C6	174.63 (18)	N3—C13—C12—C11	178.7 (2)
Zn1—N1—C1—C2	−135.94 (17)	C8—C13—C12—C11	1.3 (3)
Zn1—N1—C1—C6	43.4 (2)	C6—C1—C2—C3	0.7 (3)
C7—N3—C13—C12	−176.2 (2)	N1—C1—C2—C3	−179.9 (2)
C7—N3—C13—C8	1.5 (2)	N2—C8—C9—C10	−177.7 (2)
C7—N2—C8—C9	178.2 (2)	C13—C8—C9—C10	0.2 (3)
Zn1—N2—C8—C9	13.6 (3)	C8—C9—C10—C11	0.7 (3)
C7—N2—C8—C13	0.1 (2)	C13—C12—C11—C10	−0.3 (3)
Zn1—N2—C8—C13	−164.58 (13)	C9—C10—C11—C12	−0.7 (4)
N3—C13—C8—N2	−1.0 (2)	C1—C6—C5—C4	0.0 (3)
C12—C13—C8—N2	177.06 (18)	C7—C6—C5—C4	−176.6 (2)
N3—C13—C8—C9	−179.33 (19)	C15—N4—C14—O1	0.5 (4)
C12—C13—C8—C9	−1.3 (3)	C16—N4—C14—O1	179.4 (3)
C2—C1—C6—C5	−0.7 (3)	C1—C2—C3—C4	−0.1 (4)
N1—C1—C6—C5	−179.96 (19)	C6—C5—C4—C3	0.7 (4)
C2—C1—C6—C7	175.8 (2)	C2—C3—C4—C5	−0.6 (4)

### Hydrogen-bond geometry (Å, º)

**Table:**

*D*—H···*A*	*D*—H	H···*A*	*D*···*A*	*D*—H···*A*
N1—H1*A*···O1^i^	0.89	2.11	2.923 (2)	152
N1—H1*B*···Cl2^ii^	0.89	2.48	3.3592 (17)	170
N3—H3···O1	0.86	1.99	2.807 (2)	157
C2—H2···Cl2^ii^	0.93	2.93	3.734 (2)	145
C14—H14···Cl1^iii^	0.93	2.95	3.836 (3)	160

Symmetry codes: (i) −*x*+1/2, *y*+1/2, −*z*+1/2; (ii) −*x*+1, −*y*+2, −*z*+1; (iii) *x*+1/2, −*y*+3/2, *z*−1/2.
